# Supplementary material for: The incidence and survival after in-hospital cardiopulmonary cerebral resuscitation in end-stage kidney disease patients: A nationwide population-based study
Source: PLoS One. 2020 Aug 28;15(8):e0238029. doi: 10.1371/journal.pone.0238029 (PMC7454972; doi:10.1371/journal.pone.0238029)
Supplement: S1 Table — (DOCX) [file pone.0238029.s001.docx]

**Supplementary Table 1**. Admitted CPCR events per 1,000 admission days of admitted ESKD patients

| Year | Admitted patients | Admitted CPCR events | Admission days | Admitted CPCR incidence (95% CI) |
| --- | --- | --- | --- | --- |
| 2004 | 3,830 | 487 | 122,540 | 3.97 (3.62–4.33) |
| 2005 | 8,102 | 1,166 | 247,859 | 4.70 (4.43–4.97) |
| 2006 | 11,062 | 1,613 | 339,236 | 4.75 (4.52–4.99) |
| 2007 | 14,019 | 2,092 | 411,501 | 5.08 (4.87–5.30) |
| 2008 | 16,936 | 2,357 | 478,870 | 4.92 (4.72–5.12) |
| 2009 | 19,466 | 2,279 | 554,800 | 4.11 (3.94–4.28) |
| 2010 | 22,571 | 2,411 | 649,944 | 3.71 (3.56–3.86) |
| 2011 | 25,226 | 2,404 | 696,932 | 3.45 (3.31–3.59) |
| 2012 | 26,920 | 2,712 | 738,103 | 3.67 (3.54–3.81) |
| 2013 | 24,226 | 2,206 | 601,084 | 3.67 (3.52–3.82) |
| *P* for linear trend of Poisson regression | | | | <0.001 |

CI, confidence interval; CPCR, cardiopulmonary cerebral resuscitation; ESKD, end-stage kidney disease.
