# Supplementary material for: The incidence and survival after in-hospital cardiopulmonary cerebral resuscitation in end-stage kidney disease patients: A nationwide population-based study
Source: PLoS One. 2020 Aug 28;15(8):e0238029. doi: 10.1371/journal.pone.0238029 (PMC7454972; doi:10.1371/journal.pone.0238029)
Supplement: S2 Table — (DOCX) [file pone.0238029.s002.docx]

**Supplementary Table 2**. Median survival months

| Year of CPCR | Patients | In-hospital survivors | Proportion of in-hospital survivors (95% CI) | Median survival months after discharge [25^th^ , 75^th^ percentile] |
| --- | --- | --- | --- | --- |
| 2004 | 110 | 21 | 19.1 (12.8–27.4) | 3.0 [1.1–17.3] |
| 2005 | 373 | 82 | 22.0 (18.1–26.5) | 8.2 [1.8–50.8] |
| 2006 | 569 | 105 | 18.5 (15.5–21.9) | 9.6 [1.8–80.6] |
| 2007 | 754 | 134 | 17.8 (15.2–20.7) | 8.6 [1.0–52.0] |
| 2008 | 870 | 135 | 15.5 (13.3–18.1) | 10.7 [1.3–59.5] |
| 2009 | 835 | 169 | 20.2 (17.7–23.1) | 9.8 [1.4–47.9] |
| 2010 | 913 | 160 | 17.5 (15.2–20.1) | 9.3 [1.2–37.2] |
| 2011 | 913 | 166 | 18.2 (15.8–20.8) | 10.1 [1.8–25.7] |
| 2012 | 1,020 | 226 | 22.2 (19.7–24.8) | NA* |
| *P* trend | － | － | 0.244 | <0.001 |

CI, confidence interval; CPCR, cardiopulmonary cerebral resuscitation; NA, not available.

*The median survival months after discharge was not calculated in 2012 due to insufficient potential follow-up.
