# Supplementary material for: The incidence and survival after in-hospital cardiopulmonary cerebral resuscitation in end-stage kidney disease patients: A nationwide population-based study
Source: PLoS One. 2020 Aug 28;15(8):e0238029. doi: 10.1371/journal.pone.0238029 (PMC7454972; doi:10.1371/journal.pone.0238029)
Supplement: S3 Table — (DOCX) [file pone.0238029.s003.docx]

**Supplemental Table 3**. Summary of studies about ESKD patient post CPCR outcome

| Author and year | Patient number | Incidence rate of CPR | Outcome |
| --- | --- | --- | --- |
| Tzamaloukas, 1991(11) | 56 | NR | 20% survived to discharge |
| Moss, 1992 (10) | 74 | NR | 8% survived to discharge  3% survival in 6 months * |
| Lai, 1999 (10460936) | 24 | NR | 8.3 % survival in 1 month  0% survived to discharge |
| Lafrance, 2006 (16384828) | 24 | NR | 75% survived to discharge |
| Saeed, 2015 (12) | 56069  (NIS 2005-2011) | NR | 26.1% survived to discharge ** |
| Wong, 2015 (13) | 33731  (USRDS 2000-2010) | 1.4 / 1000 in patient days | 21.9% survived to discharge |

*Statistical significant compared to control group, 6 month survival 9% in control group (P= 0.044)

**Statistical significant compared to general population, odds ratio 1.24 (P < 0.001)

NIS: Nationwide Inpatient Sample; USRDS: US Renal Data System
