# Supplementary material for: The incidence and survival after in-hospital cardiopulmonary cerebral resuscitation in end-stage kidney disease patients: A nationwide population-based study
Source: PLoS One. 2020 Aug 28;15(8):e0238029. doi: 10.1371/journal.pone.0238029 (PMC7454972; doi:10.1371/journal.pone.0238029)
Supplement: Attachment — Submitted filename: PLOS ONE Nephrology Manuscript Review.pdf [file pone.0238029.s004.pdf]

PLOS ONE Nephrology Manuscript Review

Title: The Incidence and Survival after In-hospital Cardiopulmonary Cerebral Resuscitation in Dialytic Patients: A Nationwide Population-based Study

Authors: Chia-Hung Yang, Jia-Jin Chen, Jih-Kai Yeh, George Kuo, Cheng-Chia Lee, I-Chang Hsieh, Ming-Jer Hsieh, Ya-Chung Tian, Chih-Hsiang Chang, MD

General Comments:
This is a very interesting retrospective cohort study from Taiwan of incidence and survival after in-hospital cardiopulmonary cerebral resuscitation in ESKD patients. It identified key risk factors and key protective factors in this critical demographic. This manuscript provides an interesting look into such a well-known complication in such a high-risk population. However, the article needs to be specific in stating that they looked at ESKD population and not all dialytic patients. There are some points that need to be clearly defined. There are some grammatical errors that need to be addressed as well.

Specific comments:

Title:

- "Dialytic Patients"

This should say "end-stage kidney disease patients" because it did not include AKI patients on dialysis.

Abstract:

- "Survival rate for the first in-hospital CPCR admission"
- "Dialytic patients"

It is unclear what is meant by CPCR admission. It needs to be clearly defined. Second bullet point should say "end-stage kidney disease patients" rather than "dialytic patients" so it is clear that AKI patient on dialysis were not included.

Background:

- "Survival-to-discharge rate"
- Line 18 - Cardiac catheterization
- Line 19 – "Beta blockers,"
- Line 23 - "contradictory findings"

The first bullet point should be better defined. Did the patient receive cardiac catheterization or not? It should be better stated. Remove the comma after beta blockers. Contradictory findings should be clearly outlined and more specific.

Overall, the background is well referenced and clearly outlines the aims of the study.

Methods:

- "end-stage renal disease (ESRD)"
- "CPCR admission" under patient characteristics

Updated nomenclature favors the use of “end-stage kidney disease (ESKD).” CPCR admission needs to be clearly defined.

Overall, the authors did a good job in explaining the statistical analysis. The patient selection criteria provided a very clear overview.

Results:

- Under “Baseline characteristics”: “Regarding medication use should be reworded to say” With regards to medication use…”
- Cannot review the Additional Files because they were not provided

  Pertinent results have been clearly explained

Discussion:

- First paragraph:
  - Excellent summary of the major findings
  - The word “Statically” is misspelled. This misspelling is found in other parts of the discussion section, as well.
  - Change “Dialytic patients” to ESKD
- I found the “previous studies” paragraph to be very inciteful and interesting as compared to the findings in your study.
- “Current Study” paragraph:
  - The word “Statically” is misspelled. This misspelling is found in other parts of the discussion section, as well.
  - Change “CV” to” cardiovascular”
  - Change “QT prolong” to “QT prolongation”
  - “shown the benefits effect of beta blockers in lowing…” should be reworded to state “the beneficial effects of beta blockers in lowering…”
- As mentioned before, please be careful in using “dialysis patients.” It should clearly state ESKD patients. This is found throughout the manuscript.
- It is unfortunate that details of the cardiac arrest events were not available because that would have provided even more data.

Conclusion

- Concise conclusion
