# Supplementary material for: The incidence and survival after in-hospital cardiopulmonary cerebral resuscitation in end-stage kidney disease patients: A nationwide population-based study
Source: PLoS One. 2020 Aug 28;15(8):e0238029. doi: 10.1371/journal.pone.0238029 (PMC7454972; doi:10.1371/journal.pone.0238029)
Supplement: Attachment — Submitted filename: Response to Reviewers.docx [file pone.0238029.s005.docx]

Dear Professor Joerg Heber and reviewers,

We are pleased that you offered us an invitation to revise our work entitled “The Incidence and Survival after In-hospital Cardiopulmonary Cerebral Resuscitation in End-Stage Kidney Disease Patients: A Nationwide Population-based Study” for PLOS ONE. The comments and insights were a tremendous help to us during this revision. In the revised manuscript, in accordance with the valuable suggestions of the reviewers, we have made some modifications. The major concerns of the reviewers have been fully addressed, and the entire manuscript has been carefully revised according to the instructions as directed by PLOS ONE. We had also tried our best to address the issues of reviewer #1. All the revised words and sentences are marked as red color. We hope that the revised manuscript will retain your attention and you will judge the revised manuscript to be suitable for publication in PLOS ONE.

Thanks again for reviewing our manuscript.

Yours sincerely,

First author: Chia-Hung Yang,

Corresponding author: Chih-Hsiang Chang

Reviewer #1: This is a very interesting retrospective cohort study from Taiwan of incidence and survival after in-hospital cardiopulmonary cerebral resuscitation in ESKD patients. It identified key risk factors and key protective factors in this critical demographic. This manuscript provides an interesting look into such a well-known complication in such a high-risk population. However, the article needs to be specific in stating that they looked at ESKD population and not all dialytic patients. There are some points that need to be clearly defined. There are some grammatical errors that need to be addressed as well.

Answer: We thank for your suggestion. We have revised several sentences to make some points to be clearly defined

Reviewer #2: Outside of some minor grammatical errors and syntax, this is a well written manuscript with conclusions that are of interest to the readership of the Journal. I congratulate the authors on their findings.

Answer: Thanks for your great recommendation.
