# Supplementary material for: The incidence and survival after in-hospital cardiopulmonary cerebral resuscitation in end-stage kidney disease patients: A nationwide population-based study
Source: PLoS One. 2020 Aug 28;15(8):e0238029. doi: 10.1371/journal.pone.0238029 (PMC7454972; doi:10.1371/journal.pone.0238029)
Supplement: Attachment — Submitted filename: Response to Reviewers.docx [file pone.0238029.s006.docx]

Dear Professor Joerg Heber and reviewers,

We are pleased that you offered us an invitation to revise our work entitled “The Incidence and Survival after In-hospital Cardiopulmonary Cerebral Resuscitation in End-Stage Kidney Disease Patients: A Nationwide Population-based Study” for PLOS ONE. The comments and insights were a tremendous help to us during this revision. In the revised manuscript, in accordance with the valuable suggestions of the reviewers, we have made some modifications. The major concerns of the reviewers have been fully addressed, and the entire manuscript has been carefully revised according to the instructions as directed by PLOS ONE. We had also tried our best to address the issues of reviewer #1. All the revised words and sentences are marked as red color. We hope that the revised manuscript will retain your attention and you will judge the revised manuscript to be suitable for publication in PLOS ONE.

Thanks again for reviewing our manuscript.

Yours sincerely,

First author: Chia-Hung Yang,

Corresponding author: Chih-Hsiang Chang

Additional Editor Comments (if provided):

The authors should properly provide scientific evidence regarding the selection process and carefully describe inclusion and exclusion criteria.
Proper statistics should be performed and a flow-chart with details regarding screened and enrolled patients should be provided.

Ans: We thank for your suggestion. We have modified according to your advice.

Reviewer #3: - It is not clear why the authors excluded from the study ESKD patients who did not undergo CPCR. The analysis of the characteristics of patients who did and did not undergo CPCR after a cardiac arrest event would have been of interest and would have helped in determining the external validity of the findings (e.g., do the findings apply to all hospitalized ESKD patients who experience a cardiac arrest, or only to those who undergo CPCR?)

Ans: Thanks for your comments. In our country, only patients with Do Not Resuscitate were not performed CPCR. One of our primary goal of this study is to investigate the possible risk or protective factors of the risk of in-hospital death for these patients. Hence, we excluded the ESKD patients without undergoing CPCR.

- “We did not include CPCR events that occurred in emergency departments” the reasons for this are not clear but very likely to bias the results of the study

Ans: Thanks for your valuable comments. The study cohort was ESKD patients who experienced a first in-hospital CPCR after dialysis initiation. Because the reasons of patients with out-of-hospital cardiac arrest sometimes were not clear when coming to emergency department and the medical treatments on the ambulance were not reported in NHIRD. Therefore, the extrapolation validity was limited to ESKD patients with in-hospital CPCR. We have described this issue in the “Study cohort and design” subsection and added this to the second point of the limitations.

- “All patients were followed from their index admission until December 31, 2013” this gives the idea that patients who were alive at discharge were still followed over time, however the outcome of interest is in-hospital mortality. The authors should clarify this point, December 31 2013 seems to be the administrative censoring of the study

Ans: We agreed with your assessment. We have revised several sentences. “Patients who survived the index hospitalization with CPCR were followed from the date of discharge until December 31, 2013, or the date of death, whichever came first.”

- The authors exclude patients with a diagnosis of malignancy and those with a previous transplant although these criteria are not specified among the inclusion/exclusion criteria. Did the authors generate a statistical analytical plan drafted before data analysis?

Ans: Thanks for your comments. We revised our inclusion and exclusion criteria, as shown in the second paragraph of the “Study cohort and design” subsection. Our statistical analytical plan generally referred to the reference 21.

- The study lacks a comparison group, be it ESKD patients not undergoing CPCR or non-ESKD patients undergoing CPCR. They perform inferential statistical analyses on trends over calendar year, however this is not particularly interesting nor relevant

Ans: Thanks for your comment. In this cohort study, we focused the survival and incidence of ESKD patients with CPCR. We wanted to understand the improvement of medical care of ESKD patients with in-hospital CPCR. Figure 2 and Figure 3 show the improvement of care quality in our country over time. Therefore, the results were very important and useful for clinical doctors when discussing the medical conditions with ESKD patients and their families. Based on this, we prefer to retain this epidemiological information.

- Some of the results reported in Table 2 are puzzling: diabetes seems to be associated with better outcomes, with an OR of 0.86 with a p-value of 0.060. This is indirect suggestion of a potential selection bias in the study

Ans: We thank for your suggestion. We agree with you and added this to the second point of the limitations.

Reviewer #4: Thank you very much for the opportunity to review a manuscript titled “The Incidence and Survival after In-hospital Cardiopulmonary Cerebral Resuscitation in End-Stage Kidney Disease Patients: A Nationwide Population based Study”, which was first reviewed by other reviewers. This is the second review for this manuscript and the first review by me. This manuscript reported survival outcomes and their associated factors for patients with end stage kidney disease who received the first in-hospital cardiopulmonary cerebral resuscitation after the initiation of dialysis. The methods used appear to be valid. The finding gained also appear appropriate. Overall, this manuscript is well written. However, I have one concern. The predictors used in this study does not include important factors for outcomes of patients after cardiac arrest, which include initial arrest rhythm, witness status, and bystander status. As this study included only in-hospital cardiac arrest patients, these factors may not be important compared to out-of-hospital cardiac arrest cases. I understand that the authors briefly described this limitation in the Limitations, and I am not sure if these factors are less important for in-hospital cardiac arrest patients than for OHCA patients, but it would be better if the authors could address this issue by citing other papers. I found some typos as shown below. Therefore, it would be better if this manuscript could be proofread thoroughly again.
Ans: Thank you for this valuable comments and references. In this revision, we have added some references and changed the wording based on your suggestion in the “Study Limitations”

Line 6 under a paragraph with a heading ‘Study outcome’: What is ‘NIH’? I presume this is ‘NHI’. If not, please spell this out. The heading should be ‘Study outcomes’ as there are multiple outcomes.

The 2nd last line of the first paragraph of the Statistical analysis: A space is missing between ‘CPCR’ and ‘were’.

In the paragraph starting with ‘To our knowledge’ in the Discussion: A term ‘metaanalysis’ is less commonly used than a term ‘meta-analysis’.

In the paragraph starting with ‘The present study indicated’ in the Discussion: A clause ‘benefits effect’ sound awkward. I feel it may be ‘beneficial effects’?

Answer: Thank you for this valuable comments. We have fixed these wrong words.
